# Knowledge of, Attitude towards, and Preventive Behavior towards Tuberculosis questionnaire: translation and cross-cultural adaptation for use in Brazil

**DOI:** 10.36416/1806-3756/e20240426

**Published:** 2025-05-21

**Authors:** Rosana Maria Barreto Colichi, Sebastião Pires Ferreira, Renata Camargo Alves, Silvana Andrea Molina Lima, Hélio Rubens de Carvalho Nunes

**Affiliations:** 1. Programa de Pós-Graduação em Enfermagem, Faculdade de Medicina, Universidade Estadual Paulista Júlio de Mesquita Filho - UNESP - Botucatu (SP) Brasil.; 2. Departamento de Infectologia, Dermatologia, Diagnóstico por Imagem e Radioterapia, Faculdade de Medicina, Universidade Estadual Paulista Júlio de Mesquita Filho - UNESP - Botucatu (SP) Brasil.; 3. Faculdade Israelita de Ciências da Saúde Albert Einstein, Hospital Israelita Albert Einstein, São Paulo (SP) Brasil.

**Keywords:** Tuberculosis, Cross-cultural comparison, Attitude, Behavior

## Abstract

**Objective:**

To translate the Knowledge of, Attitude towards, and Preventive Behavior towards Tuberculosis questionnaire into Brazilian Portuguese and adapt it for use in Brazil.

**Methods:**

This methodological study followed internationally recommended guidelines for translation and cross-cultural adaptation. After permission was obtained from the original authors, the process of translation and cross-cultural adaptation began, including translation into Brazilian Portuguese by bilingual translators, synthesis of the translations, back-translation for similarity analysis, revision, and preparation of the final version. A pretest was conducted on 68 medical students.

**Results:**

Most of the questionnaire items showed strong content similarity, with minor semantic differences. The content validity index for the questionnaire was 0.882, and Cronbach’s alpha coefficients were 0.682, 0.809, and 0.613 for knowledge of tuberculosis, attitudes toward tuberculosis, and preventive behavior toward tuberculosis, respectively. Cronbach’s alpha and omega coefficients were a = 0.717, w1 = 0.673, w2 = 0.673, and w3 = 0.520.

**Conclusions:**

The process of translation and cross-cultural adaptation of the Knowledge of, Attitude towards, and Preventive Behavior towards Tuberculosis questionnaire was successful, making the Brazilian Portuguese version of the questionnaire reliable for reproducibility. It can be used in order to collect tuberculosis-related data and support changes in health education curricula.

## INTRODUCTION

Despite efforts by the WHO and many countries to eliminate tuberculosis, incidence and mortality rates have declined slowly, tuberculosis remaining a major global health concern. This situation has been worsened by COVID-19 and recent armed conflicts, creating a pressing global tuberculosis crisis. Well-structured, effective, and multisectoral responses are essential in order to meet eradication targets.^1^

In Brazil, the National Plan to End Tuberculosis as a Public Health Problem, issued in 2017, has not been able to prevent more than 80,000 new cases and approximately 5,000 deaths annually. Brazil ranks alongside nations such as Bangladesh and Zambia,^2^ reflecting gaps in tuberculosis prevention, diagnosis, treatment, and research investments.^3^

Between 2015 and 2023, tuberculosis cases increased, particularly among vulnerable groups. From 2022 to 2023, tuberculosis cases among health care workers also rose.^2^ Recognizing and preventing tuberculosis is crucial not only for patient care but also for health care professionals. 

Assessing knowledge, attitudes, and behaviors regarding tuberculosis among health students can enhance disease recognition, improve patient care, and foster empathy toward infected individuals. Findings can inform policy decisions and curriculum enhancements in health education, which remains an area requiring further research.^4^ ^-^ ^7^

The WHO developed the Knowledge, Attitudes, and Practices (KAP) questionnaire in the 1950s to assess various health issues. The tuberculosis-specific version, available since 2008, provides guidance on adapting the questionnaire to different social contexts.^8^ However, although the KAP questionnaire is effective for general knowledge assessment, it has limitations in evaluating tuberculosis-related practices and attitudes.^9^

The Knowledge of, Attitude towards, and Preventive Behavior towards Tuberculosis questionnaire, developed by Yun Choi and Geum Hee Jeong and published in English in 2018,^10^ was initially designed to assess knowledge of, attitudes toward, and preventive behaviors toward tuberculosis among Korean army soldiers. The questionnaire integrates KAP questionnaire components, features clear and concise statements, and is suitable for international studies. However, it has yet to be translated and adapted for use in Brazil. 

The objective of the present study was to translate the Knowledge of, Attitude towards, and Preventive Behavior towards Tuberculosis questionnaire into Brazilian Portuguese and adapt it for use among health students in Brazil. 

## METHODS

This study is a methodological investigation of translating the Knowledge of, Attitude towards, and Preventive Behavior towards Tuberculosis questionnaire into Brazilian Portuguese and adapting it for use in health students in Brazil. The study followed methodological guidelines from Beaton et al. and Fortes & Araújo, who proposed that self-report measures be cross-culturally adapted in accordance with international recommendations, following the steps of preparation, translation, reconciliation of translations, back-translation, revision, and pretesting.^11^ ^,^ ^12^

The original scale was based on an examination tool by the Korean Centers for Disease Control and Prevention^10^ and is multidimensional, measuring knowledge, attitudes, and preventive behavior related to tuberculosis, with original Cronbach’s alpha coefficients of 0.87, 0.83, and 0.82, respectively.^12^ To measure tuberculosis knowledge, 20 items (six of which are formulated in reverse) offer three response options (yes, no, and I don’t know)^10^ with binomial correction (correct/incorrect). The sections measuring attitudes and behavior each contain 15 items with positive statements scored on four-point Likert scales, higher scores translating to better attitudes and more appropriate behaviors toward tuberculosis. The tool offers the advantage of being evaluated by items or subcategories for each section. 

The adaptation process began with permission from the original authors. Ethical considerations were adhered to, and approval was obtained from the Research Ethics Committee of the São Paulo State University School of Medicine at Botucatu (Ruling nos. 5.278.736 and 5.453.561), in accordance with Brazilian National Health Council Resolution nos. 510/2016 and 466/2012. 

The original questionnaire was independently translated into Brazilian Portuguese by two Brazilians who were fluent in English, one of whom is in the field of health sciences and one of whom is in the field of biological sciences. The translators were identified as translator 1 and translator 2, producing two independent translations (T1 and T2). For the synthesis of the translations, a review committee was formed, including medical and nursing professionals, as well as a researcher experienced in translating and culturally adapting self-report measures. All committee members have a doctoral degree and knowledge of English and Brazilian Portuguese. The committee held meetings to compare translations and discuss until a consensus version was agreed upon (T3). 

The evaluation considered semantic equivalence, idiomatic equivalence, cultural equivalence, and conceptual equivalence. The resulting version was then back-translated into English by an independent professional who is fluent in Portuguese and English, and who had no medical training, being unaware of the original English-language version of the questionnaire. This process resulted in a back-translation (T4), which was compared with the original for similarity analysis. 

To better assess the internal consistency of the questionnaire, the committee expanded the four-point Likert scales to six points in the attitudes and behavior domains, with a minimum score of 15 and a maximum score of 90. 

For calculation of the content validity index, each item was scored on a Likert scale from 1 to 4 on the basis of the feasibility of translation to the Brazilian context.^13^ The content validity index for the entire questionnaire was calculated as the arithmetic mean of the item scores, with 100% as the approval parameter.^14^

**Figure 1:**
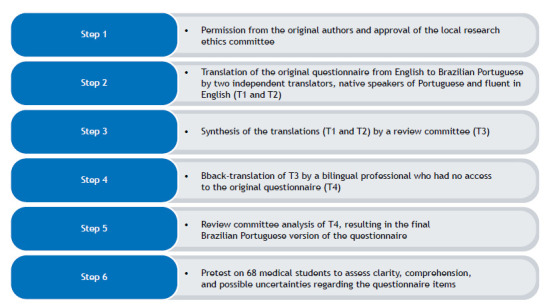
Flowchart of the process of translation and cross-cultural adaptation of the Knowledge of, Attitude towards, and Preventive Behavior towards Tuberculosis questionnaire for use in Brazil.

A pilot test was conducted on 68 medical students at a public university in Brazil. After completing the questionnaire, student judges evaluated clarity, comprehension, and possible uncertainties regarding the items, being given an opportunity to suggest improvements. On the basis of the mean of positive scores, the minimum acceptable value was set at 0.80. The entire process of translation and cross-cultural adaptation is shown in Figure 1. 

To assess internal consistency, the Kuder-Richardson formula 20 was used for knowledge-related items, whereas Cronbach’s alpha and Omega coefficients were applied with values between 0.60 and 0.95 being considered acceptable.^15^ ^-^ ^17^ Additionally, item-total correlation was analyzed, with correlations greater than 0.20 suggesting good correlation.^18^ ^,^ ^19^

## RESULTS

The process of translation and adaptation of the Knowledge of, Attitude towards, and Preventive Behavior towards Tuberculosis questionnaire occurred between May of 2023 and March of 2024. Of the 50 questionnaire items, 49 showed strong content similarity, with only minor semantic differences. 

In the process of translating the 50 items that constitute the questionnaire, similarities prevailed in 49 (98%) of the items regarding content, with few semantic differences. Only one item had exact similarity. 

For the consensus version (T3), T1 and T2 were adapted to the Brazilian culture and context, in accordance with the WHO KAP guidelines.^8^ The review committee standardized the items by using the first-person pronoun “I”. The committee opted for terms that are more commonly used by patients (“phlegm,” “tiredness,” and “fever” instead of “expectoration/sputum,” “fatigue,” and “sweating”), as well as culturally appropriate terms (“glasses and cutlery” instead of “bowls”). 

Adjustments related to the medical context were necessary because, according to the current literature, smoking is a relevant factor for greater susceptibility to tuberculosis, regardless of frequency (item 7). For the item referring to nocturnal fever, the committee opted for “nocturnal period” rather than “at dusk,” considering that patients do not always correctly identify specific times (item 11). 

To ensure clarity, the committee maintained the term “medicines” in some items to avoid repetition while preserving comprehension. Adjustments for terms commonly accepted in Brazil were necessary, such as in items 23, 24, 25, and 28 (“I think” and “I believe”). 

In the back-translation (T4), there were differences from the original because of the need to make adjustments; however, there were no conceptual errors or inconsistencies detrimental to the evaluation. The back-translation reproduced the same ideas as did the items in the original version, passing the validity checks and having no potential errors. The content validity index for the questionnaire was 0.95 in the first evaluation and 1.00 in the second, expert consensus being achieved for all items. 

A pretest was conducted in March of 2024. Most of the students to whom the questionnaire was administered were < 25 years of age (80.9%), female (57.4%), single (98.5%), and nonsmokers (91.2%). Only 7.4% of the respondents reported tuberculosis cases in their families. The mean knowledge score was 10.51, with 79% (n = 54) failing to answer 70% of questions correctly. In the attitudes domain, the mean total score was 72.3 [IQR, 20-90], being = 4.82 per item, with the mean score being lowest (2.34) for item 26. For the behavior domain, the mean total score was 61.1 [IQR, 38-87], being 4.08 per item, with the mean scores being lowest (1.57, 1.56, and 1.46) for items 42, 43, and 44, respectively. 

In the evaluation of the questionnaire, the mean coefficient calculated for clarity, comprehension, and uncertainty was 0.882. In the analysis of the internal consistency of the questionnaire, Cronbach’s alpha coefficients were 0.682 for knowledge of tuberculosis, 0.809 for attitudes toward tuberculosis, and 0.613 for preventive behavior toward tuberculosis (Table 1). With regard to Cronbach’s alpha and McDonald’s omega coefficients, the results were as follows: α = 0.717 and ω1 = 0.673; ω2 = 0.673; and ω3 = 0.520 for knowledge of tuberculosis, attitudes toward tuberculosis, and preventive behavior toward tuberculosis, respectively. The highest item-total correlations were obtained in the attitudes domain (Table 1). Chart 1 shows the original English-language version of the Knowledge of, Attitude towards, and Preventive Behavior towards Tuberculosis questionnaire and the Brazilian Portuguese version of the questionnaire. 

**Chart 1:** Original and translated versions of the Knowledge of, Attitude towards, and Preventive Behavior towards Tuberculosis questionnaire.

Original version (in English) Knowledge of, Attitude towards, and Preventive Behavior towards Tuberculosis questionnaire		Translated version (in Brazilian Portuguese) Instrumento de avaliação de conhecimento, atitudes e comportamento sobre tuberculose	
Knowledge of tuberculosis		Conhecimento sobre tuberculose	
Infection route	1-Tuberculosis can break out anywhere in the human body.	Transmissão	1-A tuberculose pode se manifestar em qualquer parte do corpo humano.
	2-Tuberculosis can be transferred through coughing and sneezing.		2-A tuberculose pode ser transmitida pela tosse e espirros.
	3-Tuberculosis may be transmitted by physical contact such as shaking hands or hugging.*		3-A tuberculose pode ser transmitida por contato físico, como aperto de mão ou abraço.
	4-Everyone infected with *Mycobacterium tuberculosis* becomes ill.*		4-Toda pessoa infectada com *Mycobacterium tuberculosis* adoece.
	5-If infected with tuberculosis once, lifelong immunity is formed.*		5-Uma vez infectado com tuberculose, se cria imunidade para o resto da vida.
	6-Tuberculosis is not transmitted through towels, plates, or bowls.		6-A tuberculose não é transmitida através de toalhas, pratos, copos ou talheres.
	7-Tuberculosis is more frequent in people who smoke a lot.		7-A tuberculose é mais frequente em pessoas que fumam.
	8-Tuberculosis is inherited by children from parents.*		8-A tuberculose é uma doença hereditária, transmitida dos pais para os filhos.
	9-Tuberculosis bacillus exists in the air.		9-O bacilo da tuberculose existe no ar.
Symptoms	10-No specific symptoms are present in the early stages of tuberculosis infection.	Sintomas	10-Não há sintomas específicos nos estágios iniciais da infecção pela tuberculose.
	11-If infected with tuberculosis, a slight fever occurs in the afternoon.		11-Quando infectado pela tuberculose, ocorre uma leve febre no período noturno.
	12-If a mild fever persists, accompanied by weight loss, tuberculosis is suspected.		12-Caso uma febre leve persistir, acompanhada de perda de peso, suspeita-se de tuberculose.
Preventive examinations	13-Chest X-rays are one way to diagnose tuberculosis.	Exames preventivos	13-O raio-X de tórax é uma forma de diagnosticar a tuberculose.
	14-Only one vaccination with BCG can provide lifelong immunity.*		14-Apenas uma vacinação com BCG pode gerar imunidade para o resto da vida.
	15-One should be examined if a prolonged cough with sputum persists for more than 2 weeks.		15-Deve-se examinar se a tosse prolongada com catarro persistir por mais de 2 semanas.
	16-Even if no special symptoms of coughing or sputum are present, I should be examined for tuberculosis if I have weight loss, fatigue, and so on.		16-Mesmo que não haja sintomas como tosse ou catarro, eu deveria ser examinado para a tuberculose se tiver perda de peso, cansaço, etc.
Treatment	17-Tuberculosis cannot be treated in the absence of overt symptom.*	Tratamento	17-A tuberculose não pode ser tratada na ausência de sintomas evidentes.
	18-Tuberculosis is treated by taking medicine every day for at least 6 months.		18-A tuberculose é tratada tomando remédios todos os dias por pelo menos 6 meses.
	19-One can recover from tuberculosis if medical treatment is followed, but if not, death can result.		19-Pode-se recuperar da tuberculose se o tratamento médico for seguido, mas, se não for, pode resultar em morte.
	20-Treatment is difficult, and if anti-tuberculosis drugs are not taken regularly, drug resistance can occur.		20-O tratamento é difícil e, se os remédios não forem tomados regularmente, pode ocorrer resistência da bactéria aos medicamentos.
Attitudes towards tuberculosis		Atitudes em relação à tuberculose	
Recognition of tuberculosis	21-I do not mind if friends or people close to me know about my tuberculosis infection.	Reconhecimento da tuberculose	21-Não me importo se amigos ou pessoas próximas a mim souberem da minha infecção por tuberculose.
	22-If I get tuberculosis diagnosis, I should immediately inform the army.		22-Se eu receber o diagnóstico de tuberculose, devo informar imediatamente as pessoas com quem trabalho e convivo.
	23-I think that tuberculosis can be caught without even realizing it.		23-Eu acredito que a tuberculose pode ser contraída sem perceber nada.
	24-I think that I may experience obstacles in my familial and professional life if I am infected with tuberculosis.		24-Eu acho que posso enfrentar problemas na minha vida familiar e profissional se estiver contaminado com tuberculose.
	25-I think that tuberculosis is a very serious disease.		25-Eu acho que a tuberculose é uma doença muito grave.
	26-I have a higher than usual likelihood of tuberculosis infection.		26-Tenho uma probabilidade maior do que o normal de contrair tuberculose.
Preventive examinations	27-I think that it helps to prevent tuberculosis if I get a tuberculosis medical examination regularly every year.	Exames preventivos	27-Eu penso que realizar exame médico periódico para a tuberculose ajuda na prevenção dessa doença.
	28-I think that one should be examined for tuberculosis if there is a tuberculosis patient among one’s family or friends.		28-Eu acho que eu deva ser examinado para tuberculose caso um familiar ou amigo esteja com essa doença
	29-I think that it is not too difficult to get a tuberculosis checkup in the military if one has symptoms of tuberculosis.		29-Penso que não é muito difícil fazer um exame de tuberculose nas unidades de saúde se alguém tiver sintomas da doença.
Treatment	30-If I am diagnosed with tuberculosis, I will take an anti-tuberculosis drug steadily for at least 6 months under a doctor’s direction.	Tratamento	30-Se eu for diagnosticado com tuberculose, tomarei os medicamentos regularmente por pelo menos 6 meses sob supervisão de profissionais da saúde.
	31-If a friend discontinues taking an anti-tuberculosis medication, I will persuade the friend to take anti-tuberculosis medication continuously.		31-Se um amigo interromper o uso de medicamentos antituberculose, eu o convencerei a tomar os remédios de forma contínua.
	32-I will encourage tuberculosis patients around me to get treatment.		32-Eu incentivarei os pacientes com tuberculose ao meu redor a receberem tratamento.
	33-I think that tuberculosis can be cured completely if detected and treated early.		33-Eu penso que a tuberculose pode ser curada completamente se detectada e tratada o quanto antes.
Preventive education	34-I am interested in tuberculosis.	Educação preventiva	34-Tenho interesse em tuberculose.
	35-I think that education about tuberculosis is needed.		35-Eu acho que é necessária a educação sobre tuberculose.
Preventive behavior towards tuberculosis		Comportamento preventivo em relação à tuberculose	
Infection route	36-I try not to spend for a long time in places where air does not circulate well, such as Internet cafes, karaoke establishments etc.	Transmissão	36-Procuro não ficar muito tempo em locais onde o ar não circula bem, como bares ou estabelecimentos semelhantes etc.
	37-If I expectorate or spit out sputum, I wrap it in tissue paper and throw it out.		37-Se eu tossir com catarro, faço isso em um lenço de papel e o jogo no lixo.
	38-When I sneeze or cough, I cover my mouth.		38-Quando espirro ou tusso, cubro minha boca.
	39-In order to maintain fresh air indoors, I often open windows or find another way to ventilate the space.		39-Para manter o ar fresco em ambientes fechados, geralmente abro as janelas ou encontro outra maneira de ventilar o espaço.
	40-I cover my mouth with a handkerchief or tissue when coughing.		40-Cubro minha boca com um lenço de papel ao tossir.
Preventive examinations	41-If a cough lasts more than 2 or 3 weeks, I go to the army medical corps or an army hospital to get a checkup.	Exames preventivos	41-Se a tosse durar mais de 2 ou 3 semanas, procuro uma unidade de saúde para fazer exames.
	42-I obtain a chest X-ray regularly every year.		42-Eu faço um raio-X de tórax regularmente todos os anos.
Preventive education	43-I frequently read materials designed to raise awareness about tuberculosis.	Educação preventiva	43-Com frequência, leio materiais informativos sobre tuberculose.
	44-I actively participate in education about tuberculosis.		44-Eu participo ativamente da educação sobre tuberculose.
Healthy lifestyle	45-If I suffer from stress, I have ways of dealing with it.	Estilo de vida saudável	45-Se eu sofrer de estresse, tenho maneiras de lidar com isso.
	46-I do not smoke for health reasons.		46-Eu não fumo por motivos de saúde.
	47-I usually eat well-balanced meals to maintain good health.		47-Costumo fazer refeições balanceadas para manter uma boa saúde.
	48-I do not eat excessively because doing so harms the immune system and overall health.		48-Eu não como excessivamente, pois isso prejudica o sistema imunológico e a saúde em geral.
	49-I am sure to wash my hands after going out or exercising.		49-Tenho certeza de que lavo as mãos depois de sair ou praticar atividades físicas.
	50-I usually engage in regular exercise to maintain good health.		50-Eu costumo praticar atividade física regularmente para manter uma boa saúde.

*Reverse questions.

**Table 1:** Cronbach’s alpha coefficient for evaluation of a Brazilian Portuguese version of the Knowledge of, Attitude towards, and Preventive Behavior towards Tuberculosis questionnaire.

Item	Scale mean if item deleted	Scale variance if item deleted	Corrected item-total correlation	Cronbach’s alpha if item deleted
1	10.37	10.177	0.233	0.674
2	9.58	10.095	0.323	0.668
3	9.93	9.797	0.231	0.674
4	9.79	9.289	0.451	0.649
5	9.96	9.559	0.308	0.665
6	10.37	10.419	0.115	0.683
7	9.97	10.393	0.038	0.697
8	9.60	10.517	0.079	0.685
9	10.06	9.784	0.235	0.674
10	10.21	9.380	0.425	0.652
11	10.24	9.700	0.321	0.664
12	10.19	9.644	0.319	0.664
13	9.75	10.313	0.093	0.688
14	10.06	9.905	0.195	0.678
15	9.78	9.661	0.319	0.664
16	10.16	9.533	0.346	0.661
17	10.07	9.464	0.346	0.661
18	10.06	9.875	0.205	0.677
19	9.61	10.180	0.231	0.674
20	9.60	9.941	0.377	0.663
21	67.85	105.142	0.005	0.838
22	66.84	92.197	0.587	0.787
23	67.56	96.698	0.363	0.803
24	67.59	96.962	0.347	0.804
25	67.16	94.645	0.585	0.789
26	69.97	105.372	0.019	0.832
27	68.19	94.605	0.329	0.808
28	67.18	91.342	0.642	0.783
29	68.25	94.668	0.392	0.801
30	66.65	93.008	0.777	0.781
31	66.78	92.980	0.648	0.785
32	66.57	94.666	0.709	0.785
33	66.91	96.440	0.506	0.794
34	68.22	94.772	0.423	0.798
35	66.60	93.736	0.787	0.782
36	57.78	53.843	0.357	0.574
37	57.04	50.013	0.476	0.546
38	55.67	62.133	0.127	0.610
39	56.01	60.803	0.173	0.606
40	57.37	59.571	0.077	0.629
41	56.49	60.314	0.130	0.612
42	59.87	59.936	0.145	0.610
43	59.88	61.379	0.097	0.615
44	59.99	60.591	0.159	0.607
45	57.01	59.833	0.088	0.624
46	56.51	55.436	0.201	0.607
47	56.46	54.707	0.430	0.566
48	57.12	52.198	0.483	0.552
49	56.76	54.700	0.359	0.575
50	56.30	56.910	0.293	0.588

## DISCUSSION

The present study described the process of developing a Brazilian Portuguese version of the Knowledge of, Attitude towards, and Preventive Behavior towards Tuberculosis questionnaire for undergraduate health students, following the methodological steps recommended in the literature. The translated questionnaire achieved semantic, idiomatic, conceptual, and cultural equivalence, with adequate comprehension by the target population and good internal consistency. 

The use of international scales facilitates comparisons of studies across countries, expanding opportunities for successful interventions. However, cross-cultural adaptation of instruments requires a meticulous process involving multiple stages that address both textual and technical aspects.^20^ This demands methodological rigor to maintain the reliability and validity of the original instrument.^21^

The Consensus-based Standards for the Selection of Health Measurement Instruments initiative recommends that translated versions of assessment tools undergo review by a committee of experts and pretesting to improve the selection of instruments for measuring outcomes in research and clinical practice, thus contributing to the development of assessment tools that are more appropriate.^22^

Although the Knowledge of, Attitude towards, and Preventive Behavior towards Tuberculosis questionnaire is a comprehensive, 50-item questionnaire, its concise, objective, and easy-to-understand sentences facilitated the process of translating it into the target language. Backward translation ensured equivalence between the translations and the original English-language questionnaire. 

In making necessary adaptations to the local culture, the expert committee compared translations without major controversies or disagreements among members, consistently considering scientific evidence on the subject^23^ and focusing on effective communication by selecting the terms that are most commonly used by patients and laypeople in general,^24^ aiming for an approach aligned with the Brazilian reality. The clear and detailed completion guidelines appear to have been fundamental to data collection. Similar results can be found in the literature, highlighting the importance of pretesting, given that this stage can identify potential difficulties in completing, applying, or understanding the items.^25^

Because the Knowledge of, Attitude towards, and Preventive Behavior towards Tuberculosis questionnaire was originally developed with the use of simple language, our study sought to expand its use. We examined each item in detail and its appropriateness within any scope of practice and determined that no items needed to be excluded. The pretest results indicated that the participants understood the questionnaire well. 

Although Cronbach’s alpha remains the most commonly used reliability index in research, several other aspects of test capability should be considered, including validity evidence, intercultural fairness, and practicality. The limitations of Cronbach’s alpha have been discussed elsewhere,^17^ with better alternatives including omega coefficients, which offers advantages for applied research in which items differ in quality or have skewed distributions. Omega coefficients can extend the usefulness of alpha coefficients in estimating the reliability of internal consistency scores on a multidimensional scale with smaller samples,^17^ such as the one evaluated in the present study. 

In general, most of the students in our sample did not have adequate knowledge of tuberculosis at the time of questionnaire administration, despite their attitudes and behaviors being considered positive. Our results regarding tuberculosis knowledge are similar to those obtained in Korea, where the instrument was developed (11.64; 58.2%), with low recognition of populations more vulnerable to tuberculosis, as well as low scores on questions related to behavior, especially those concerning examinations and preventive education.^10^

In a scenario in which tuberculosis continues to be a neglected disease, translating and adapting a tuberculosis questionnaire fulfills the objective of providing an instrument that can enable comparisons and identification of gaps in teaching, as well as the implementation of policies to ensure that tuberculosis becomes an integral part of any educational curriculum for health professionals. Given the widespread need to promote public policies that guarantee continuous improvement of care and effectiveness of measures to control and prevent tuberculosis, it is essential to include tuberculosis education in the curricula of future health professionals.^26^

The absence of experts from other regions of the country in the evaluation may have been a limiting factor in the present study, given that Brazil is a large, culturally diverse country. Future studies should focus on the application of the final version of the questionnaire, its psychometric validation, and its use in other health professionals . 

The recommendation to use internationally standardized instruments is aimed at improving research quality and enabling more reliable comparisons between countries and regions. Our translation and cross-cultural adaptation of a questionnaire for assessing knowledge, attitudes, and behaviors regarding tuberculosis offers an important resource for researchers in Brazil by providing a version translated and revised by an expert committee, and subjected to pretesting and statistical analysis in accordance with international guidelines. The use of the translated questionnaire can contribute to collecting information on tuberculosis and support changes in health education curricula. 
